# Eating Quality of Ribeye Steaks From Young Bulls and Steers Is Comparable: A Study Examining the Effect of Sex, Frame Size, and Feed Time on Palatability Attributes

**DOI:** 10.1002/fsn3.70359

**Published:** 2025-06-30

**Authors:** Yunus Khatri, Elisabeth Huff‐Lonergan

**Affiliations:** ^1^ School of Science Engineering and Technology RMIT University Ho Chi Minh City Vietnam; ^2^ Department of Animal Science Iowa State University Ames Iowa USA

**Keywords:** eating quality, feed time, frame size, ribeye steaks, steers, young bulls

## Abstract

The eating quality of older bull beef in the United States has been considered inferior due to its tough texture, undesirable flavor, and color, often being relegated to manufactured meat items. However, the potential to use young bulls was the focus of this research as the need for alternative protein sources increases. The aim of this study was to investigate the palatability attributes of beef ribeye (*Longissimus thoracis et lumborum*) steaks from young bulls and steers of three frame sizes (small, medium, and large), fed three time periods (180, 210, and 240 days) prior to slaughter. Animals (135 bulls and 135 steers) of an approximate slaughter age of 15 months were utilized. Mean palatability attribute scores were significantly different, albeit small, between bulls and steers—tenderness (6.8 versus 7.1), connective tissue (6.8 versus 7.0), juiciness (6.5 versus 6.8), flavor (6.6 versus 6.8), flavor intensity (6.6 versus 6.8) as well as myofibrillar (2.61 kg/cm^2^ versus 2.23 kg/cm^2^), and residual shear force (2.51 kg/cm^2^ versus 2.18 kg/cm^2^) measures. Such differences may not be discernable to consumers. No significant palatability difference was found for time on feed for the sexes; however, it significantly increased intramuscular lipid content up to 240 days though not perceived in tenderness scores. Small‐framed sized animals were significantly more tender when compared to other frames. This article provides evidence using a trained panel, that meat from young bulls is comparable to their steer counterparts. Thus, the potential to use young bulls as a source for sub primal cuts holds promise for increasing our protein supply.

## Graphical abstract

Palatability of ribeye steaks from young bulls and steers of 15 months age were comparable. Tenderness, juiciness, and beef flavor scores were similar with small‐framed animals being rated the highest scores.

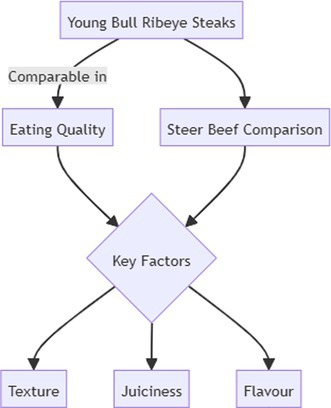

## 1 Introduction

Projections from the Food and Agricultural Organization (2017) are that the demand for meat from ruminants will maintain an increase at the rate of 1.5% per year. Predictions of robust meat demand in Western countries of 15%–71% have been reported by Jia et al. (2023). Red meat is renowned for its high‐quality protein as well as for its essential nutrients (Henchion et al. 2017). Lean beef provides high biological value protein (Górska‐Warsewicz et al. 2018) and is an important source of essential amino acids (Wolfe et al. 2018), B‐group vitamins, omega‐3 polyunsaturated fatty acids, conjugated linoleic acid, iron, phosphorus, and zinc (McAfee et al. 2010). However, the negative effects on human health of cholesterol and fatty acids in meat have raised concern (Singh et al. 2003) but more recently Harcombe (2019), Dehghan et al. (2017) and others have contested such findings. With estimates approaching 10 billion people on Earth by the year 2050 (FAO 2018; World Bank 2019), humankind faces a daunting challenge to feed a world population with twice its current protein requirement. New protein sources are being sought and the option to use young bulls is most certainly one way of achieving a varying source of protein in the diet.

Debates have been raised on water and land use as well as greenhouse gas (GHG) emissions from animal production, some of which have been fervent at times (Miller et al. 2022; Laestadius et al. 2014; Torrella 2023). To add to this dilemma, meat consumption and health has also been controversial (Johnston et al. 2019; Farvid et al. 2021; Mozaffarian 2020; WHO 2015). Numerous authors have reported that food production at the farm stage represents 61% of food's GHG emission of which livestock production, contributes 65% of these emissions (Scarborough et al. 2023; Crippa et al. 2021; Rosenzweig et al. 2020; Poore and Nemecek 2018; Mariantonietta et al. 2018). Food‐system emissions have been reported to amount to 18 Gt CO_2_ equivalent per year globally, representing 34% of total GHG emissions in 2015. The largest contribution came from agriculture and land use/land‐use change activities (71%). Note: CO_2_ is the most important GHG, but agriculture is also a large source of methane and nitrous oxide (Grossi et al. 2019). In order to capture all GHG emissions from food production, researchers express them in kilogram equivalents of carbon dioxide (Ritchie and Dowlatabadi 2018).

The systematic review by Titterington et al. (2022) provides information on a number of factors leading to dangerous interactions with cattle by humans. These authors report that animal temperament can be affected by genetics, farm management practices, and environment. Bulls, in particular, are physically strong and can inflict injuries on humans inadvertently and/or through aggressive behavior. Age of the animal has also been reported to affect animal aggression with younger bulls being less likely to attack (Sheldon et al. 2009). Castration is commonly used as a method to render the animal more docile (Stafford and Mellor 2005), improve carcass quality in terms of tenderness, marbling etc. (Dudley 2017), reduce unwanted mating (Hinch and Lynch 1987), and furthermore reduce dark‐cutting in beef (Tarrant 1981). However, welfare matters over the intense pain and distress caused by castration and cortisol levels after castration (Stafford and Mellor 2005; Marquette et al. 2023) has led to growing public concern and increased intervention from bovine veterinarians in a number of countries relating to the methods and treatments employed (Coetzee et al. 2010).

It is well documented that young bulls exhibit higher production efficiencies (growth rate and feed conversion ratios) resulting in a larger reduction in GHG emission intensities when compared to steers (Samsonstuen et al. 2020; McGee et al. 2023). Furthermore, reducing slaughter age has been a key factor in decreasing emission intensities even further. Findings by Teagasc (2022) have shown that there was little difference in tenderness and overall liking of striploins from bulls slaughtered between 15 and 22 months of age.

In concert with the above, Davison et al. (2020) and Henchion et al. (2017) provide innovative paradigms that meat has an important role to play as part of a sustainable diet and as a contributor to food security. This premise is supported by a growing wealth of research data utilizing anti‐methanogenic legumes, microalgae, and rumen microbial manipulation, paving the way to significant reductions in methane emissions from ruminants (Huws et al. 2023; Davison et al. 2020). We, as authors of this publication, also promulgate a reduction in red meat consumption and at the same time recommend a balanced sustainable diet.

The eating quality of meat is dependent on both pre‐ and post‐slaughter factors. Historically, consumers consistently rated tenderness as the most important sensorial attribute in the palatability of beef (Corbin et al. 2015). With further research, it has been revealed that flavor has become the most important attribute (49% to 55%) of beef eating satisfaction compared to tenderness (36% to 40%) (Legako et al. 2015; Chail et al. 2017; McKillip et al. 2017). O'Quinn et al. (2016) noted that tenderness, flavor, and juiciness accounted for 43.3%, 49.4%, and 7.4%, respectively, of the variation in overall liking of beef palatability from 1800 consumer observations. In particular, the eating quality of beef is dependent upon all three factors—tenderness, juiciness, and flavor—as well as the interaction among these traits (O'Quinn et al. 2018). Tenderness of beef is multidimensional and has been extensively reviewed. It is affected by, among other components, sex, breed, age, and cross‐linking of collagen, nutrition plane, stress, electrical stimulation, postmortem aging, hanging methods, as well as temperature and sarcomere length (Miller 2004; Hopkins 2004; Nunes et al. 2015). Intramuscular fat (IMF), commonly termed marbling, also plays a role in the tenderness of meat (Warner et al. 2021). In the review on tenderness in the aforementioned article, varying correlation coefficients from very low negative *r* = −0.06 to good approaching strong *r* = 0.68 (Schober et al. 2018) are provided between tenderness and IMF. Marbling can be controlled through genetics (Albrecht et al. 2011), nutrition (Pethick et al. 2004), and management (Park et al. 2018). The leptin gene is primarily responsible for intramuscular fat deposition (Bonnet et al. 2007) and is a valuable genotype (DeVuyst et al. 2007). Mechanisms for sensory tenderness have been postulated by Smith and Carpenter (1976) explaining reduced resistance to breakdown and higher IMF leading to a softened perception of toughness.

When comparing the palatability attributes of bulls and steers, variable results have been reported. A number of studies (Jacobs et al. 1977; Nogalski et al. 2018; Moran et al. 2017) have shown that meat from bulls has been rated as less tender than that of steers. Due consideration must also be given to toughness as a result of age (Schoonmaker et al. 2002; Joseph and Connolly 1974) as well as the effect of postmortem aging and dietary regimen (Luiz et al. 2019) which can have a marked effect on tenderness. Chriki et al. (2012) identified variability in meat tenderness using muscle characteristics, biochemical traits as well as a trained sensory panel. Using a cluster analysis, these researchers determined the muscle characteristics of importance for beef tenderness. In addition to the lowest shear force values for the most tender meat, muscles in the highest tender cluster had the lowest total and insoluble collagen contents, the highest mitochondrial enzyme activity (isocitrate dehydrogenase), the highest proportion of slow oxidative muscle fibers, the lowest proportion of fast‐glycolytic muscle fibers, and the lowest average muscle fiber cross‐sectional area, which includes intramuscular fat.

Since consumption of bull meat is fairly common in many parts of Europe, greater emphasis has been placed by producers on feed efficiency, increased muscle mass and weight to enable better utilization of feed from an improved sustainability perspective (Jia et al. 2023). Bull beef tends to be leaner than their castrated counterparts with higher muscling yields and better feed conversion ratios assisting in cost reduction for the farmer (Cafferky et al. 2019). The consumption of bull meat in some markets necessitates finishing animals on high concentrate diets (Moran et al. 2017). Further work by Moran et al. (2020) showed that age of young bulls between 16 and 19 months had a relatively small influence on tenderness. A relatively recent study by Nakitari (2021), demonstrated that young bulls and steers of 11 months showed no significant difference for tenderness of aged meat at 1°C for 21 days using Warner‐Bratzler shear force (WBSF) measures and myofibril fragmentation index (MFI). These reseachers recommended that it would be possible to place such animals in one category in a classification scheme and also help to avoid castration of bulls.

The comparison of young bulls and steers has been the focus of this study. The potential to use young bulls as a table meat source holds promise for increasing our protein supply. The objective of this study was to compare the sensory attributes of *Longissimus thoracis et lumborum* meat steaks of various grades, from young bulls and steers of three frame sizes, fed for three different time periods following a 14‐day aging period. To the best of our knowledge, there is little information available providing such comprehensive comparisons.

## 2 Materials and Methods

This study was approved by the Institutional Review Board of Iowa State University. Written informed consent was obtained from all study participants. Ethics Committee approval was received under authority Meat Lab number 09/1992/MO.

### 2.1 Source of Materials

A total of 270 yearling bulls and steers were born and raised (without implants) at three Iowa State University farms. As calves, one‐half were randomly castrated and allotted at random to one of three slaughter dates with equivalent frame size representation, maintaining an approximately equivalent age of 15 months for each group of cattle at the time of slaughter. Frame size was visually assessed by a five‐member panel of experienced evaluators as described in the US standards for grades of feeder cattle (USDA 1979). Three frame sizes were used; cattle of each frame size were of similar genetic makeup and environmental influence (Reiling et al. 1992). Small‐framed cattle were 1/4 Jersey, 1/4 Angus, and 1/2 initial female. Medium‐framed cattle were 1/8 Jersey, 1/4 Angus, 1/8 Simmental, and 1/2 initial female. The large‐framed cattle consisted of 1/4 Angus, 1/4 Simmental, and 1/2 initial female. The initial female of these three synthetic lines of cattle developed at Iowa State University consisted primarily of crossbred cows as a result of a dairy‐beef crossbreeding project.

Cattle were fed an 80% concentrate ration (on a dry‐matter basis) and straw *ad libitum* after weaning at approximately 6 months. The animals were serially slaughtered between mid‐June and mid‐August with a 30‐day interval between each slaughter date. Feed times corresponded to 180, 210, and 240 days on feed.

### 2.2 Carcass Treatments

Cattle were slaughtered at a commercial packing plant in Des Moines, Iowa. After approximately 24 h chill period at 4°C, carcasses were quartered between the twelfth and thirteenth thoracic vertebrae. USDA yield and quality grades were determined by three trained personnel from Iowa State University and one USDA grader.

Samples of *Longissimus thoracis et lumborum* muscle were excised as described by Goñi et al. (2007), fabricated into two equal 2.5 cm portions from the right‐hand side of the carcass. Each cut was individually wrapped, labeled, and shipped on ice under refrigerated conditions (4°C) to Iowa State University Meat Laboratory where they were deboned and each muscle sample was vacuum packed (Dixiepac, 100), in polyamide, polyethylene PA/PE‐70 bags (Cryovac) and aged at 2°C for 14 days prior to freezing at −18°C (Ren et al. 2021). Colle et al. (2016) found that an aging period of 14 days was commonly used for optimizing beef eating quality, relating to a plateau in proteolysis tenderization with minor or little improvement in tenderness beyond such a period.

### 2.3 Proximate Analysis

A thin sliver at the surface of the 12th and 13th rib was removed for proximate analysis. These samples were denuded by dissecting the intermuscular and subcutaneous fat. Prepared strips were frozen and pulverized for 30 s in liquid nitrogen with a Waring blender. Samples were packaged in polyethylene bags and stored at −18°C until required. The percentages of moisture and fat were determined on powdered muscle samples, in triplicate, by oven drying and petroleum ether extraction, respectively (AOAC 1987).

### 2.4 Sensory Evaluation

Meat preparation, cooking, sensory training, and sensory evaluation were followed as described in a previous paper (Khatri and Huff‐Lonergan 2023). Steaks were thawed for 48 h at 2°C and then broiled to an internal temperature of 70°C. The internal temperature of steaks was monitored at the geometric center using copper–constantan thermocouples attached to an Omega Digital Trendicator. Steaks were broiled to an internal temperature of 40°C, turned, and then broiled further to a final temperature of 70°C. Once removed from the broiler, steaks were individually wrapped in aluminum foil, placed in a 60°C warming oven, removed, and cut into approximately 1.27 × 1.27 × 3.18 cm sample cubes. Two samples randomly chosen were served to an 8‐ to 10‐member trained sensory panel. Panelists were individually seated in booths in a room separated from the preparation area and provided with water and plain crackers to cleanse their palates between samples. Red fluorescent lights were used to eliminate any biases due to the color of the samples.

Panelists (seven males and three females aged between 22 and 43 years) were trained in accordance with the guidelines provided by the American Meat Science Association (2015). All individuals were beef eaters (at least four times a week) from the Department of Animal Science and Food Science and Nutrition. Participants, although previously trained, received six 2‐h training sessions in a 2‐week period immediately preceding testing. During each training session, there was both discussion and sensory assessment of representative samples using the following sensory definitions: Myofibrillar tenderness—a measure of how easily the sample breaks down into smaller pieces after three chews; Connective tissue—the amount of insoluble connective tissue that remains in the mouth after thorough chewing; Juiciness—the sensation of free fluids released from the meat upon chewing; Flavor intensity—a measure of the perceived intensity of beef flavor; and Flavor—a measure of the sensation perceived as beef flavor. Scores for tenderness (T), connective tissue (CT), juiciness (J), flavor intensity (Fl), and flavor (F) were based on an 8‐point descriptive scale (tenderness 1 = extremely tough, 8 = extremely tender; connective tissue, 1 = abundant, 8 = none; juiciness, 1 = extremely dry, 8 = extremely juicy; flavor intensity, 1 = extremely bland, 8 = extremely intense; flavor, 1 = extremely off flavor, 8 = extremely flavorful). All participants were able to achieve repeatable evaluations and were thus selected for this analysis. Values for each sensory attribute of a steak were collected on paper ballots and were reported as an average of panelist scores.

### 2.5 Warner‐Bratzler Shear Force Measurements

On completion of each sensory evaluation session, six 1.3 cm diameter cores were removed from three sections (central, medial, lateral) of cooked steaks that had been tempered to room temperature (25°C). Cores were removed parallel with the axis of the muscle fiber and used for WBSF assessment using the same conditions reported in the article by Khatri and Huff‐Lonergan (2023). WBSF measurements were carried out using the lnstron Universal Testing Machine, model 5542, (lnstron Corp. Canton, MA) with a V‐shaped blade on a WBSF attachment and a 50‐kg load cell at crosshead speed of 100 mm/min. Shear force deformation curves were recorded by means of a chart recorder using a chart speed of 100 mm/min. Each core was sheared twice, perpendicular to the fiber direction as described by Culler et al. (1978). The twelve values were then averaged. Curve readings were divided into two parts as recommended by Moller (1981). The first break point was recognized as the compressive/myofibrillar force relating to the myofibrillar component of tenderness while the second yield point relates to the residual force of the connective tissue component of tenderness. Values for each steak are reported in kg/cm^2^.

### 2.6 Statistical Analysis

Data were analyzed as a completely randomized design utilizing a 2 × 3 × 3 factorial arrangement with the procedure GLM of SAS (1985). An ANOVA was conducted to evaluate the fixed effects of sex (bulls and steers), frame size (small, medium, and large), feed time (180, 210, and 240 days), and the interactions (sex × frame, sex × feed time, frame × feed time) to determine the eating quality by the least squares method. Three‐way interactions were not significant and were removed as a source of variation. Post hoc analysis using Tukey's test was carried out to determine differences for all significant effects, which included sensory panel data, marbling group, and shear force measures. Pearson correlation coefficients were calculated between the sensory attributes and other parameters (moisture, lipid content, myofibrillar force, and residual force) using the CORR procedure of SAS. Unless stated otherwise, all tests are reported as significant using *α* = 0.05.

## 3 Results and Discussion

Overall carcass data on the bulls and steers slaughtered are presented in Table 1. Bull carcasses had significantly lower (*p* < 0.05) marbling scores, fat thickness, and greater (*p* < 0.05) hot carcass weights and ribeye areas when compared to steer carcasses. These findings are consistent with previously reported work (Devlin et al. 2017; Nogalski et al. 2018; Blanco et al. 2020). Koch et al. (2018) posit that feedlot animals have greater growth rates when compared to pasture‐fed animals and are harvested younger, regardless of final body weight. Cafferky et al. (2019) obtained some interesting results when determining whether sire breed (Aberdeen Angus, Belgian Blue, Charolais, Hereford, Limousin, Salers, Simmental, and Parthenaise) and/or castration had an effect on meat quality of *M. longissimus thoracis et lumborum* from crossbred bulls and steers in addition to their relationship on traits such as WBSF, intramuscular fat%, drip loss%, ultimate pH, and color. The feeding regime was monitored with all animals being finished to a specified carcass conformation and fat score range. Bulls and steers were slaughtered at approximately 478 (+/− 24) days and 634 (+/− 52) days, respectively. Vacuum‐packaged steaks were aged for 2 or 14 days at 4°C depending on the trait being determined. Their findings showed that sex had a significant effect (*p* < 0.05) on WBSF values, with bull samples having higher shear force values compared to those of steers. Breed, however, had no effect on WBSF measures. The highest‐scoring bulls deposited approximately half the amount of intramuscular fat% compared to those of steers, with Aberdeen Angus sired meat samples having the highest amount of marbling while Belgian Blue and Parthenaise sired offspring had the least. Thus, castration increased intramuscular fat deposition, facilitated by increased lipid uptake and lipogenesis and reduced lipolysis (Bong et al. 2012). Bulls had the highest cook loss%, with Limousin‐reared samples having the lowest cook loss values. All these traits play an important role in the overall eating experience for consumers.

**TABLE 1:** Selected overall means and standard error of parameters for bulls and steers.

Parameter	Bulls	Steers	SEM^a^
Number	135	135	
Hot carcass wt (kg)	362.2^x^	322.2^y^	2.969
Marbling Score^b^	944^y^	1017^x^	6.412
Fat thickness (mm)	7.5^y^	11.3^x^	0.241
Ribeye area (cm^2^)	32.0^x^	29.7^y^	0.495

*Note:* ^x–y^Means within the same row bearing different superscripts are significantly different (*p* < 0.05).

^a^ SEM = is the pooled standard error of the means.

^b^ 900 = Slight^0^, 1000 = Small^0^.

Palatability attributes for bulls and steers given in Table 2 show values with very small numerical differences. Bulls ranked significantly lower (*p* < 0.05) than steers for all palatability attributes measured, while Warner‐Bratzler myofibrillar force and Warner‐Bratzler CT (residual) force were significantly higher (*p* < 0.05). Juiciness, flavor, and flavor intensity were significantly higher (*p* < 0.05) for steers compared to bulls. Studies conducted by Roeber et al. (2000) have shown that consumers often tend to generalize sensory traits and may wrongly evaluate traits in favor of another trait, such as being more flavorful if the cut is tender, termed the halo effect and hence, one specific reason to employ trained panelists. Despite statistically significant values reported herein for palatability, these differences may not necessarily be discernible by consumers.

**TABLE 2:** Means and standard error of means for overall palatability and shear force attributes of *Longissimus thoracis et lumborum* muscle from bulls and steers.

Parameter	Bulls	Steers	SEM^a^
Tenderness	6.8^xy^	7.1^y^	0.031
Connective tissue	6.8^y^	7.0^x^	0.027
Juiciness	6.5^y^	6.8^x^	0.031
Flavor	6.6^y^	6.8^x^	0.026
Flavor Intensity	6.6^y^	6.8^x^	0.029
Myofibrillar force (kg/cm^2^)	2.61^x^	2.23^y^	0.042
Residual force (kg/cm^2^)	2.51^x^	2.18^y^	0.039

*Note:* ^x–y^Means within the same row bearing different superscripts are significantly different (*p* < 0.05).

^a^ SEM is the pooled standard error of the least square means.

Analysis of variance for bulls and steers conducted for carcass and palatability traits, listing individual sources of variation, are given in Supporting Information S1. Sex, frame size, and time on feed were highly significant (*p* < 0.01) for fat thickness, lipid content, and moisture. Sex and frame size were significant for marbling score, tenderness, CT, residual force, and fat thickness; time on feed being significant (*p* < 0.05) for moisture content and sex being significant (*p* < 0.05) for juiciness, flavor, and flavor intensity. All remaining two‐way interactions were not significant. The treatments used in this experiment were sex (bulls verses steers), frame size (small, medium, and large), and time on feed (180, 210, and 240 days). Young bulls have advantages over steers in terms of growth rate, food conversion efficiencies, and leanness of meat (Sinclair et al. 1998) while steers being castrated are slower muscle producers as they lack testosterone responsible aiding the process of protein accretion (Venkata et al. 2015). Frame size is a determinant of muscle mass, with small animals producing small muscle sizes. However, smaller frame sized cattle have a propensity to deposit fat more quickly (Cafferky et al. 2019) which in turn provides for well‐marbled steaks. Further discussion is provided below.

Tenderness, juiciness, and flavor are key consumer attributes in the acceptance of palatable beef steaks (Legako et al. 2015). However, these attributes can vary depending on a number of factors including breed of cattle, sex, feeding regime, length of feeding, stress, muscle type, pre‐, and post‐slaughter processing. Tenderness can be measured instrumentally but is not an accurate sensory indicator of acceptability for consumers. Flavor has been rated as the highest sensorial property in the palatability of beef steaks by consumers (O'Quinn et al. 2024), much of the flavor compounds being present in fat, and in this study intramuscular fat. Juiciness also plays a key role in the eating quality of beef and the combination of these factors is examined based on sex, time on feed, and frame size.

Due to the highly significant influence of sex in the model, individual sexes were analyzed using frame size, time on feed, and frame size × time on feed interaction as sources of variation. Supporting Information S2 represents the analysis of variance for *Longissimus thoracis et lumborum* muscle traits of bulls. Frame size was significant for lipid content, marbling score, and moisture content. Time on feed was found to be significant (*p* < 0.05) for lipid content. Frame size × time on feed interaction was highly significant (*p* < 0.01) for CT scores only. All remaining effects were non‐significant (*p* < 0.05). As with bulls, a similar breakdown based on frame size, time on feed, and frame size × time on feed effect was made in Supporting Information S3. Steers showed highly significant effects (*p* < 0.01) based on frame size for fat thickness, lipid content, marbling score, moisture, tenderness, CT (*p* < 0.01) myofibrillar force, and residual force. Longer time on feed was highly significant (*p* < 0.01) for fat thickness, lipid content, marbling scores, and moisture content. All remaining effects were non‐significant (*p* < 0.05).

When viewing frame size (Table 3) bulls showed a significant difference in hot carcass weight from small to large frame. Steers also showed a similar trend. Marbling scores for bulls varied significantly (*p* < 0.05) between small and large‐framed carcasses and declined with an increase in size. Steers also significantly decreased (*p* < 0.05) in marbling score with an increase in frame size but the differing effect was only between the small‐framed and the rest of the carcasses. These results are in line with findings reported by Tatum et al. (1982). This greater propensity to fatten for small‐framed animals has been well established (Dolezal and Tatum 1983; Belk et al. 1991). Pethick et al. (2004) and Tatum et al. (1982) explained that the growth rate of small and large‐framed cattle reaches its maximum up until they reach physiological maturity, that is, the stage of growth when fattening commences. Small‐framed cattle reach that maturity point at an earlier age and at a lighter weight than do large‐framed cattle (Block et al. 2001). As the small‐framed cattle slow down in growth, they begin to fatten as the large‐framed cattle continue growing for a while longer (Cianzio et al. 1982). Intramuscular fat is late maturing, but as shown by Pethick et al. (2004), it does not mean that fat accretion within intramuscular adipocytes is late maturing. Improvements in marbling score have been shown to increase tenderness and flavor which is now considered the most important trait in eating satisfaction (Corbin et al. 2015; O'Quinn et al. 2024). Fat thickness did not significantly differ (*p* < 0.05) between frame sizes for bulls while ribeye areas increased significantly (*p* < 0.05) based on increase in frame size. In contrast, fat thickness for steers decreased significantly (*p* < 0.05) with increase in frame size while differences in ribeye area remained non‐significant.

**TABLE 3:** Descriptive statistics (means and standard error of means) of selected carcass parameters for small‐, medium‐, and large‐framed bulls and steers.

Parameter	Bulls				Steers			
	Small	Medium	Large	SEM^a^	Small	Medium	Large	SEM^a^
Number	45	44	46		45	46	44	
Hot carcass wt. (kg)	295.7^z^	337.7^y^	375.8^x^	8.227	289.2^z^	319.8^y^	358.3^x^	5.921
Marbling score^a^	967.0^x^	942.0^xy^	924.0^y^	16.008	1054.0^x^	1002.0^y^	997.0^y^	20.312
Fat thickness (mm)	8.5	8.0	7.5	0.732	13.3^x^	12.0^y^	9.25^z^	0.886
Ribeye area (cm^2^)	30.3^z^	32.3^y^	33.8^x^	1.050	28.9	29.7	29.7	0.680

*Note:* ^x–z^Means within the same row, for each sex group, bearing different superscripts are significantly different (*p* < 0.05).

^a^ SEM = is the pooled standard error of the means.

Time on feed for both bulls and steers (see Table 4) showed a significant increase in hot carcass weight. This hot carcass weight increase seems to be in part, associated with a significant increase in fat thickness for steers while increased ribeye area among bulls may account for weight gains too. Increases in weight based on feed time for backfat and ribeye area have been reported in previous work by Joseph and Connolly (1974) and Block et al. (2001). Testosterone is reported to adhere to receptors within muscle, raising the level of amino acid incorporation into protein and increasing the protein accretion and growth rate (Venkata et al. 2015). Given a ruminant's genetic capacity for protein accretion, Meehan et al. (2021) studied protein ratios in ruminants and showed that lean‐body mass can be enhanced and carcass composition manipulated provided a rumen undegradable protein (digested and absorbed in the intestine, Manoukian et al. 2021) is fed. Rapidly growing animals have a greater ratio of protein synthesis to protein degradation than slower growing animals (Bulle et al. 2007). Marbling deposition did not show any significant changes for bulls with increased feed time. On the other hand, steers demonstrated a significant increase in marbling with time on feed between 180 and 240 days. Our findings corroborate previously reported evidence (Sami et al. 2004; Dolezal and Tatum 1983; Belk et al. 1991) of significant increases in marbling deposition for steers with increases in time on feed. May et al. (1992) reported that marbling increased curvilinearly with days on feed being between 0 and 196 days.

**TABLE 4:** Descriptive statistics (means and standard error of means) of selected carcass parameters for bulls and steers stratified on the basis of days on feed.

Parameter	Bulls				Steers			
	180	210	240	SEM^a^	180	210	240	SEM^a^
Number	45	44	46		45	46	44	
Hot carcass weight (kg)	305.0^z^	339.2^y^	359.5^x^	6.442	301.7^z^	324.1^y^	358.3^x^	5.680
Marbling score^a^	942.0	951.0	944.0	14.545	999.0^y^	1011.0^xy^	1043.0^x^	18.074
Fat thickness (mm)	7.0^y^	8.2^xy^	8.5^x^	0.551	8.4^z^	12.0^y^	13.8^x^	0.775
Ribeye area (cm^2^)	30.2^y^	32.3^x^	33.4^x^	0.740	29.1	29.7	29.9	0.645

*Note:* ^x–z^Means within the same row, for each sex group, bearing different superscripts are significantly different (*p* < 0.05).

^a^ SEM = is the pooled standard error of the means.

Data of chemical, sensory and Warner‐Bratzler shear force measurements of *Longissimus thoracis et lumborum* muscle are provided in Table 5.

**TABLE 5:** Least square mean values and standard error of means for certain chemical, sensory, and shear force measurements of *Longissimus thoracis et lumborum* muscle of bulls and steers stratified on the basis of frame size.

Parameter	Bulls				Steers			
	Small	Medium	Large	SEM^a^	Small	Medium	Large	SEM^a^
Intramuscular lipid (%)	4.38^x^	3.29^y^	3.53^y^	0.202	5.91^x^	4.79^y^	4.45^y^	0.238
Moisture (%)	72.92^y^	73.84^x^	73.79^x^	0.213	71.15^y^	72.13^x^	72.39^x^	0.029
Tenderness	6.9	6.8	6.8	0.055	7.2^x^	6.9^y^	6.9^y^	0.055
Connective tissue	6.9	6.9	6.8	0.049	7.1^x^	6.7^y^	6.9^y^	0.022
Juiciness	6.5	6.6	6.5	0.055	6.8^x^	6.7^xy^	6.6^y^	0.055
Flavori intensity	6.6	6.6	6.6	0.049	6.8	6.7	6.7	0.032
Flavor	6.6	6.5	6.5	0.047	6.8	6.7	6.8	0.055
Myofibrillar force (kg/cm^2^)	2.62	2.56	2.55	0.078	2.05^x^	2.31^y^	2.32^y^	0.071
Residual force (kg/cm^2^)	2.45	2.43	2.51	0.069	1.98^y^	2.26^x^	2.30^x^	0.068

*Note:* ^x–y^Least square means within the same row, for each sex group, bearing different superscripts are significantly different (*p* < 0.05).

^a^ SEM is the pooled standard error of the least squares mean.

There was a significant difference (*p* < 0.05) between small‐framed and medium‐ and large‐framed animals when considering intramuscular lipid values. Small‐framed animals had significantly lower (*p* < 0.05) moisture levels than their counterparts. Sensory panel and Warner‐Bratzler data for bulls stratified on the basis of frame size revealed no significant difference for every parameter tested. Steers exhibited a different pattern, and this may be related to lipid content. Tenderness and CT scores were significantly higher (*p* < 0.05) for the small‐framed animals than their large‐framed contemporaries. This difference was also verified by the myofibrillar and residual forces from Warner‐Bratzler peak force measurements. Marbling has a positive impact on beef eating quality, affecting tenderness, juiciness, and flavor (O'Quinn et al. 2024). Flavor and flavor intensity were higher for steers when compared to bulls, though not significant (*p* > 0.05). Flavor is a very complex attribute influenced by numerous factors both pre‐ (age, diet, genetics, marbling level, and treatment/handling) and post‐slaughter (conditioning, cooking, storage, and display) (O'Quinn et al. 2024). Heating of meat brings about the development of flavor and odor in beef. The taste buds are stimulated by water soluble substances emanating from precursor complexes and register a response that is perceived in the brain. Odor is perceived when volatile compounds bind to receptors in the olfactory bulb behind the nasal cavity and stimulate a response (Brewer 2006). Juiciness scores also differed significantly in favor of small‐framed animals, probably due to the higher fat content. Scientists in the USA (Dikeman 1987; Tatum 1981) have maintained that a value of approximately 3% ether‐extractable lipid is necessary to provide juicy, tender beef.

Least square means for certain chemical, sensory, and Warner‐Bratzler shear force measurements of *Longissimus thoracis et lumborum* muscle stratified on the basis of days on feed for bulls is laid out in Table 6. Lipid content increased with time on feed but only significantly between 180 days and 210 days, and 210 days, and 240 days for bulls and steers respectively. Aoki et al. (2001) found extended feeding of cattle to heavier weights (500 kg) did not increase in intramuscular fat beyond a carcass weight of about 420 kg; a ‘maximum value’ concept was proposed of declining feed intake as animals reach their mature body size and the carcass composition does not change. Results from this study showed increases in intramuscular fat% for all frame size groups based on feed time except for small‐framed bulls fed for 240 days. Pethick et al. (2004) state that metabolic modifiers which increase muscle growth such as hormonal growth promotants, β‐agonists, and organic chromium can reduce the rate of intramuscular fat accretion. The genetic potential of the cattle should also be considered (Chen et al. 2019). Animals exhibiting high‐marbling potential possess up‐regulated levels of mRNA associated with adipogenesis, and reduced mRNA levels linked to myogenesis. Intramuscular fat accretion differences between breeds may be due to differences in energy metabolism and the synthesis of fatty acids in liver, as well as the expression of regulatory genes (Wang et al. 2017). Thus, suitable sire selection is extremely important for high‐marbling beef production (Park et al. 2018).

**TABLE 6:** Least square mean values and standard error of means of certain chemical, sensory, and shear force measurements of *Longissimus thoracis et lumborum* muscle of bulls and steers stratified on the basis of days on feed.

Parameter	Bulls				Steers			
	180	210	240	SEM^a^	180	210	240	SEM^a^
Intramuscular lipid (%)	3.12^y^	4.02^x^	4.03^x^	0.202	4.27^y^	4.85^y^	5.99^x^	0.238
Moisture (%)	73.87^x^	73.18^y^	73.48^y^	0.213	72.35^x^	72.04^x^	71.30^y^	0.038
Tenderness	6.9	6.9	6.8	0.055	7.1	7.1	7.0	0.055
Connective tissue	6.8	6.9	6.9	0.049	7.0	7.0	7.1	0.002
Juiciness	6.6	6.6	6.5	0.055	6.8	6.7	6.7	0.055
Flavor intensity	6.7	6.6	6.6	0.049	6.7	6.7	6.7	0.032
Flavor	6.5	6.6	6.5	0.047	6.7	6.7	6.7	0.055
Myofibrillar force (kg/cm^2^)	2.56^xy^	2.21^y^	2.70^x^	0.078	2.25	2.25	2.07	0.071
Residual force (kg/cm^2^)	2.51	2.52	2.64	0.069	2.17	2.22	2.17	0.068

*Note:* ^x–y^Least square means within the same row, for each sex group, bearing different superscripts are significantly different (*p* < 0.05).

^a^ SEM is the pooled standard error of the least square means.

Moisture values decreased significantly (*p* < 0.05) coinciding with increases in lipid level. The inverse relationship between intramuscular fat and moisture has been reported by several authors (Legako et al. 2015; Jeremiah et al. 2002). The current study results are in concert with previous findings, although the correlations (see Tables 11 and 12) are not as high as reported by Legako et al. (2015) obtaining *r* = −0.92.

No significant palatability difference (*p* < 0.05) could be reported between feed times among bulls and steers. Warner‐Bratzler myofibrillar force and residual force values were also non‐significant for steers (*p* < 0.05). On the other hand, bulls showed significantly higher (*p* < 0.05) values for myoflbrillar force between 210 and 240 days on feed. Residual force was not significantly (*p* < 0.05) affected. Research feed trials used varying lengths of time on feed found that longer feeding periods for cattle tended to decrease taste panel acceptability (Aoki et al. 2001; Tomkins et al. 2006). It is interesting to note from this study that tenderness and juiciness scores were slightly reduced at 240‐day feeding time. In addition, a study by Zurbriggen et al. (2022) found aging and fat thickness to play an important role in tenderness. Using ultrasound measurements, Zurbriggen et al. (2022) extended the feeding time period in four lots (64, 91, 119, and 147 days on feed) to achieve a minimum of 8.0 mm subcutaneous fat on Angus and Hereford × Angus non‐implanted steers. They excised the *Longissimus thoracis* muscle between the 10th and 12th rib, vacuum aged 2.5 cm cuts at 5°C for a period of 3 and 14 days. Their results indicate a decrease in ribeye area and increase in fat thickness with the increasing finishing period. Bondurant et al. (2016) reported similar finding and our work in this current study is consistent with these findings for steers. The 8.0 mm endpoint was sufficient to achieve suitable feed efficiency and obtain tender meat, especially when aged for 14 days. From a cost perspective, feeding steers beyond 8.0 mm would result in a negative economic impact under the type of system examined. Dolezal et al. (1982) fed steers for 100–230 days and found that extending time on feed beyond 100 days provided little additional palatability assurance. Our work concurs with the aforementioned findings.

Table 7 provides a breakdown of certain chemical and carcass measurements for bulls and steers based on time on feed and frame size. The feed trial was an extended feeding trial that was formed as part of a project involving ultrasound and other performance measures; feeding periods of 240 days are atypical and double the usual time in the USA. Marbling scores indicate no significant (*p* > 0.05) difference between large‐framed animals irrespective of slaughter period. However, bulls showed an increase in marbling as they peaked at 210 days followed by a reduction in the degree of marbling when bulls were fed 240 days. It is possible that marbling for the small‐framed bulls cannot be increased beyond its genetic potential (Bruns, n.d.) when fed 240 days in this study. Small‐framed bulls fed for 210 days showed significantly greater (*p* < 0.05) amounts of marbling than large‐framed animals.

**TABLE 7:** Least square mean values and standard error of means for selected carcass measurements of *Longissimus thoracis et lumborum* muscle from bulls and steers based on frame size and time on feed.

Parameter	Sex	Groups^a^									
		11	12	13	21	22	23	31	32	33	SEM^b^
Marbling^c^	Bull	961^vw^	947^vw^	917^w^	985^v^	941^vw^	922^w^	953^vw^	936^w^	935^w^	16.10
	Steer	1040^wxy^	978^z^	981^z^	1046^wx^	990^yz^	996^xyz^	1072^w^	1022^wxyz^	1029^wxyz^	20.0
Fat thickness (mm)	Bull	7.0^wx^	8.3^vwx^	6.6^x^	9.6^v^	7.5^vwx^	8.1^vwx^	9.0^vw^	9.2^v^	7.8^vwx^	0.86
	Steer	10.8^wxy^	8.4^xy^	6.3^z^	12.8^vw^	11.6^vwx^	8.1^xy^	15.9^u^	13.6^uv^	11.9^vw^	0.90
Lipid content (%)	Bull	3.74^wxy^	3.01^yz^	2.69^z^	4.96^v^	3.12^xyz^	3.92^wxy^	4.33^vw^	3.71^wxy^	4.04^vwx^	0.350
	Steer	4.89^vwxy^	4.03^y^	3.92^y^	5.84^uv^	4.59^wxy^	4.16^xy^	6.88^u^	5.73^uvw^	5.29^vwx^	0.274
Moisture (%)	Bull	73.28^wx^	73.89^vw^	74.38^v^	72.35^x^	73.89^vw^	73.38^vwx^	73.16^wx^	73.74^vw^	73.57^vw^	0.369
	Steer	71.85^xy^	72.61^x^	72.56^x^	71.32^xy^	72.23^x^	72.56^x^	70.38^y^	71.57^vy^	72.04^xy^	0.301

*Note:* ^u–z^Least square means within the same row having different superscripts are significantly different (*p* < 0.05).

^a^ Group designations = 10 × Time on feed (1 = 180 days, 2 = 210 days, 3 = 240 days) + frame size (1 = small, 2 = medium, 3 = large).

^b^ SEM is the pooled standard error of the least square means.

^c^ 900 = Slight^0^, 1000 = Small^0^.

There was no significant difference (*p* > 0.05) for subcutaneous fat thickness between small and medium‐framed bulls from the 180‐day feed period and medium and small bulls from 210‐ and 240‐day phases, respectively. Fat thickness measurements were significantly different (*p* < 0.05) between large‐framed animals during the first slaughter period and small‐, medium‐, and large‐framed animals from slaughter periods following 210‐ and 240‐day feeding time. There was also a significant difference (*p* < 0.05) between large‐framed bulls fed 180 days and small‐framed bulls fed for 210 and 240 days, respectively. These large‐framed bulls had a 6.6 mm fat thickness, a value that could bring about cold induced toughness (Tatum et al. 1982; Zurbriggen et al. 2022). However, the sensory panel result for tenderness of these bulls scored an average of 6.7 out of an 8 point scale (see Table 8). Belk et al. (1991) reported that subcutaneous fat tended to shift from the ventral portions of the carcass toward the dorsal regions as fattening progressed in steers. Reports from Camfield et al. (1997) and Grona et al. (2002) have shown that large‐framed cattle produce larger carcasses lower in fat.

**TABLE 8:** Least square mean values and standard error of means for palatability and shear force measurements of *Longissimus thoracis et lumborum* muscle from bulls and steers based on frame size and time on feed.

Parameter	Sex	Groups^a^									
		11	12	13	21	22	23	31	32	33	SEM^b^
Tenderness	Bull	6.9	6.9	6.7	6.9	6.7	6.9	6.9	6.8	6.7	0.096
	Steer	7.3^x^	7.0^yz^	7.0^yz^	7.1^yz^	7.0^yz^	7.0^yz^	7.3^x^	7.0^yz^	6.9^z^	0.090
Connective tissue	Bulls	6.9^xy^	7.1^x^	6.6^z^	6.9^xy^	6.8^xy^	7.1^x^	7.0^xy^	7.0^xy^	6.9^xy^	0.086
	Steers	7.2^x^	6.9^y^	6.9^y^	7.1^xy^	6.9^y^	6.9^y^	7.2^x^	7.0^xy^	6.9^y^	0.080
Juiciness	Bulls	6.6^xy^	6.8^x^	6.6^xy^	6.3^y^	6.6^xy^	6.6^xy^	6.4^y^	6.4^y^	6.4^y^	0.095
	Steers	6.9^x^	6.7^y^	6.7^y^	6.7^y^	6.7^y^	6.8^y^	6.8^y^	6.7^y^	6.6^y^	0.090
Flavor intensity	Bulls	6.6^xy^	6.8^x^	6.3^y^	6.6^xy^	6.5^y^	6.7^xy^	6.6^xy^	6.5^xy^	6.5^xy^	0.082
	Steers	6.9	6.6	6.9	6.7	6.8	6.8	6.8	6.7	6.7	0.010
Flavor	Bull	6.5^xy^	6.5^xy^	6.2^y^	6.7^x^	6.6^xy^	6.6^xy^	6.5^xy^	6.4^xy^	6.6^xy^	0.157
	Steer	6.8	6.8	6.8	6.7	6.7	6.7	6.8	6.7	6.6	0.080
Myofibrillar force (kg/cm^2^)	Bull	2.50^xy^	2.51^xy^	2.59^xy^	2.51^xy^	2.42^y^	2.52^xy^	2.67^xy^	2.61^xy^	2.78^x^	0.137
	Steer	2.08^xy^	2.28^xy^	2.31^xy^	2.07^xy^	2.38^x^	2.32^xy^	2.01^y^	2.29^xy^	2.33^xy^	0.011
Residual force (kg/crn^2^)	Bull	2.33^y^	2.41^xy^	2.53^xy^	2.41^xy^	2.42^xy^	2.42^xy^	2.62^xy^	2.53^xy^	2.72^x^	0.120
	Steer	2.06^y^	2.22^x^	2.23^x^	2.00^y^	2.32^x^	2.31^x^	1.90^y^	2.20^x^	2.39^x^	0.113

*Note:* ^x–z^Least square means within the same row having different superscripts are significantly different (*p* < 0.05).

^a^ Group designations = 10 × Time on feed (1 = 180 days, 2 = 210 days, 3 = 240 days) + frame size (1 = small, 2 = medium, 3 = large).

^b^ SEM is the pooled standard error of the least square means.

Large variations in intramuscular lipid content were seen between the bulls. Large‐framed animals fed for the 180‐day period did not differ significantly (*p* > 0.05) in intramuscular lipid from medium‐framed bulls in both feeding periods 180 and 210 days. Significantly lower (*p* < 0.05) values were also observed for intramuscular lipid contents between bulls of medium‐framed size fed 180 days, and those of small and large frame fed 240 days. Small‐framed bulls also had the highest amount of intramuscular lipid within each feed period. It has been thought that marbling is a late developing tissue. With a greater emphasis being put on selecting genetics that preponderate toward marbling and/or lean muscle growth, it is important to understand that marbling development is taking place earlier than what was previously thought, commencing at around six months after conception and continuously grows through the lifecycle of the animal (Nguyen et al. 2021).

Moisture values were not significantly different (*p* > 0.05) between small and medium‐framed bulls from the 180‐day feeding phase and those of medium and large‐framed bulls in the 210‐day feed trial, as well as all animals in the 240‐day feed trial. A significant (*p* < 0.05) moisture difference is also noted for large‐framed bulls from the 180‐day feed phase and small‐framed bulls from the 180‐day and 240‐day feeding periods, respectively.

Moving onto steers, small‐framed steers fed for the longest duration (Table 7) had the highest marbling scores and were significantly different (*p* < 0.05) from medium and large‐framed steers fed for 180 days. Also, small‐framed steers from the 180‐day period had marbling scores that were not significantly different (*p* > 0.05) from the 240‐day period irrespective of frame size or that of small‐framed steers fed for 210 days. Such an extended feeding phase is clearly unwarranted for improvements in marbling for small‐framed steers (Block et al. 2001; Aoki et al. 2001). Values for fat thickness among steer carcasses were significantly different (*p* < 0.05) between small‐framed steers fed for the 240‐day period and all other treatments except medium‐framed animals fed the 240‐day term. As described previously above with the large‐framed bulls fed for 180 days, the fat cover for large‐framed steers was also low at 6.3 mm with an average sensory panel tenderness score of 7.0 (see Table 8). This would suggest, although not actually determined, that cold induced toughness may not have occurred or could have been nullified due to the 14‐day aging period (Locker 1985; Harper 1999). Medium‐framed steers fed the full 240‐day period were not significantly different (*p* > 0.05) from those carcasses within the same feed group as well as small‐ and medium‐framed animals in the slaughter group fed 210 days.

Lipid values for small‐framed steers from the 240‐day feed period were not significantly different (*p* > 0.05) from steers of the same frame size from 210‐day feed period and medium‐framed steers fed for 240 days. Large‐framed steers fed for 240 days were not significantly different (*p* > 0.05) from small steers fed for 180 days, and medium‐framed steers fed 210 days, respectively. No significantly difference (*p* < 0.05) could be reported for small and medium steers fed 180 days, and those fed 210 days of large frame. Moisture values for small‐framed steers were lower than medium and large‐framed counterparts, although a significant difference (*p* < 0.05) was detected between the small and medium steers from the 180‐day and 240‐day feed periods and this is a likely consequence of fat deposition (Gredell et al. 2018; Cafferky et al. 2019). No significant difference (*p* < 0.05) was detected among all steers in the 180‐day feed phase, medium‐ and large‐framed steers in the 210 days and large‐framed steers in the 240‐day feed period. The inverse relationship between moisture and fat has been well established (Gredell et al. 2018). Compositional analysis has shown that protein levels are relatively constant in a proximate analysis, excess protein is converted to urea and excreted, fat and moisture levels being more dynamic (Beermann 1994) as shown here.

It is evident from the palatability data and shear force of bulls summarized in Table 8 that no significant differences (*p* > 0.05) existed for tenderness among bulls. Thus, the extended feed time would not be recommended in this case. Tenderness of beef is influenced by genetics, plane of nutrition, aging, sex, age of animal, pre‐ and post‐slaughter care, and their inter‐relationship (Warner et al. 2010). The process by which meat becomes tender is rather complex. The elements that alter the myofibrillar/cytoskeletal proteins and their interactions affect the architecture and integrity of the skeletal muscle cell, thereby rendering the meat tender through degradation and oxidation (Huff‐Lonergan et al. 2010). Various authors (Camfield et al. 1997; Cafferky et al. 2019) provided substantive evidence regarding the increase in tenderness with the increase in feed time. However, the 14‐day aging process used in this present study is considered optimal (Colle et al. 2016; Lucero‐Borja et al. 2014) for beef eating quality, relating to a plateau in proteolysis tenderization with minor or little improvement in tenderness beyond such a period. CT scores varied little among bulls except between large‐framed bulls of the 180‐day period and remaining carcasses. Medium and large‐framed carcasses from 180‐ and 210‐day feed trials also showed no significant difference (*p* > 0.05) in CT. The remaining carcasses did not differ significantly (*p* < 0.05) on the basis of CT. Juiciness scores varied significantly (*p* < 0.05) between medium‐framed bulls fed 180 days, and all those bulls fed full term.

Beef flavor is developed during heating, as degree of doneness enters into play, with most of the flavor components formed by the Maillard reaction and heat lipid denaturation (Kerth and Miller 2015). Intramuscular fat plays a major contributory factor in flavor development as volatile flavor compounds are soluble in fat and their dynamic release is perceived later than for water soluble compounds (Guichard 2002). Beef flavor intensity scores were higher (*p* < 0.05) for medium bulls of the 180‐day feed period than large‐framed bulls of the same period. Flavor differences were perceived only between large‐framed bulls of the 180‐day feed time and small‐framed bulls of the 210‐day feed time. Myofibrillar shear force values differed among bulls for those of medium frames fed 210 days and that of large framed fed 240 days. Residual force measurements varied significantly (*p* < 0.05) between bulls of small frame fed 180 days and that of large frame fed 240 days.

Palatability and Warner‐Bratzler shear force measurements for steers based on frame size and time on feed are also reported in Table 8. A significant difference (*p* < 0.05) in tenderness is reported between small‐framed steers fed 180 and 240 days with that of large‐framed steers fed 240 days. In addition, the aforementioned small‐framed steers were rated significantly (*p* < 0.05) higher than all remaining counterparts. Furthermore, small‐framed steers from 180‐ and 240‐day feed periods differed significantly for CT from the remainder, except for small‐ and medium‐framed steers fed 210 and 240 days, respectively. Large‐framed steers fed 240 days were equivalent in tenderness to medium‐ and large‐framed bulls irrespective of slaughter period; this may be attributed to the maturity of the animals; however, we did not assess maturity as part of this study. Higher juiciness scores (*p* < 0.05) were noted for small steers from the 180‐day feed frame when contrasted with all other remaining steers, regardless of frame size and time on feed. The sensory panel was unable to detect any differences (*p* > 0.05) in flavor and flavor intensity from steaks from these steers. Warner‐Bratzler myofibrillar force determinations were not significantly different (*p* > 0.05) except for medium‐framed steers from the 210‐day feed period and small‐framed steers of the 240‐day feed phase. Except for small‐framed steers that did not differ significantly (*p* > 0.05) in terms of residual force measurement and the significant difference (*p* < 0.05) between small‐framed steers and large‐framed steers fed 240 days, no differences were apparent. Johnson et al. (1990) reported that bulls aged 6 days postmortem had higher shear force values than steers, but at 13 days postmortem, equivalent shear force values were obtained.

More recent evidence (Anaruma et al. 2020) as well as the consensus view from many authors over the years (Cross et al. 1973; Jacobs et al. 1977) is that young entire males are of comparable eating quality to that of their castrated counterparts and that toughening due to collagen is only significant when extremes of age are compared. Results reported in this work substantiate previous work in terms of high scores for bulls and their general acceptability in terms of eating quality. In addition, tenderness scores have improved over the many years due to genetic breeding, plane of nutrition, age at slaughter, conditioning of meat as well as general pre‐ and post‐slaughter care. These factors have contributed to the desire for more flavorful beef due to the consistent tenderness experienced in beef eating quality (O'Quinn et al. 2024).

A breakdown of palatability and shear force data based on marbling is provided in Tables 9 and 10 for bulls and steers, respectively. No significant palatability difference (*p* < 0.05) between bulls could be reported except for juiciness, flavor intensity and myofibrillar force for Modest and Moderate degrees of marbling. Steers also exhibited a similar pattern whereby a significant difference (*p* < 0.05) could be detected for those muscles between the slightly abundant degree of marbling and the remainder of marbling categories for tenderness, CT, myofibrillar, and residual force. The high degree of marbling had a significant (*p* < 0.05) impact in terms of flavor between Traces and slightly abundant groupings. Though not significant in this study, the juiciness and flavor scores increased with degree of marbling particularly at the modest level. These results are agreement with previous studies by Tatum et al. 1980) and Corbin et al. (2015). Residual force values for steers varied but declined with a concomitant increase in marbling. Significant differences (*p* < 0.05) were obtained between the categories of traces and slight and that of modest, moderate, and slightly abundant. The number of cattle falling into the marbling categories are low in some cases; however, these are actuals and are representative of the feeding trial. Repeat experimentation may be required to substantiate these numbers further. As indicated above, no significant differences could be detected for the marbling categories in relation to tenderness (except for slightly abundant IMF, though only one sample) and this is evidenced by the low correlations between marbling and tenderness, also cited by Parrish (1974) and Dikeman (1987) (*r* = 0.138 and 0.111 for bulls and steers, respectively from our study).

**TABLE 9:** Least square mean values for palatability and shear force attributes of *Longissimus thoracis et lumborum* muscle from bulls stratified on the basis of marbling category.

	Marbling category				
	Traces	Slight	Small	Modest	Moderate
Number	26	85	20	3	1
Tenderness	6.7	6.9	6.9	7.1	6.6
Connective tissue	6.8	6.9	6.9	7.1	6.7
Juiciness	6.4^xy^	6.6^xy^	6.6^xy^	6.8^x^	6.1^y^
Flavor	6.6^x^	6.6^x^	6.6^x^	6.8^x^	6.1^y^
Flavor intensity	6.6	6.5	6.6	7.0	6.5
Myofibrillar force (kg/cm^2^)	2.68^xy^	2.66^xy^	2.43^xy^	1.95^y^	2.85^x^
Residual force (kg/cm^2^)	2.60	2.55	2.44	2.29	2.03

*Note:* ^x–y^Least square means within the same row bearing different superscripts are significantly different (*p* < 0.05).

**TABLE 10:** Least square mean values for palatability and shear force attributes of *Longissimus thoracis et lumborum* muscle from steers stratified on the basis of marbling category.

	Marbling category					
	Traces	Slight	Small	Modest	Moderate	Slightly abundant
Number	5	63	60	2	4	1
Tenderness	6.9^y^	7.0^y^	7.1^y^	7.1^y^	7.1^y^	7.5^x^
Connective tissue	6.9^y^	7.0^y^	7.1^xy^	7.1^xy^	7.1^xy^	7.6^x^
Juiciness	7.0	6.7	6.7	6.8	6.8	6.8
Flavor	6.9	6.8	6.7	6.7	6.8	6.8
Flavor intensity	6.5^y^	6.7^xy^	6.8^xy^	6.6^xy^	6.9^xy^	7.1^x^
Myofibrillar force (kg/cm^2^)	2.45^y^	2.36^y^	2.13^y^	2.10^y^	2.32^y^	1.67^x^
Residual force (kg/cm^2^)	2.34^x^	2.35^x^	2.14^xy^	1.93^y^	1.89^y^	1.51^z^

*Note:* ^x–z^Means within the same row bearing different superscripts are significantly different (*p* < 0.05).

Consumer palatability ratings for strip loins varying in marbling levels and quality grades were evaluated by Corbin et al. (2015). These authors found that prime steaks were rated as more tender (*p* < 0.05) than high choice, top choice, select, and standard but were not significantly different (*p* > 0.05) from low choice steaks. Standard grade steaks were less tender (*p* < 0.05) than all higher rated marbling steaks and not significantly different (*p* > 0.05) from select. Juiciness ratings received positive associations with fat content increasing with fat%, prime steaks being juicier (*p* < 0.05) than all other steaks of lower fat content. Choice steaks were juicier (*p* < 0.05) than standard and select samples. There was no significant difference (*p* > 0.05) between standard and select steak samples. Prime steaks were rated higher (*p* < 0.05) for flavor liking than all other treatments except low choice. All choice steak categories were rated more likable (*p* < 0.05) for flavor than select and standard grades. Corbin et al. (2015) posit that a background beef flavor is similar across the USDA quality grades, with fat% and fat‐like flavor being the overwhelming key to consumer flavor liking. Since standard and select steaks scored much lower for flavor liking, this same working group went further using trained panelists to differentiate flavor liking specifically stating that fat‐like and umami flavors were much more desirable and in larger quantities in higher USDA quality grades.

Due to confounding effects of sex, age, and time on feed, caution is required when discussing and concluding from the results given for correlations herein. Jiu et al. (2020) split their discussion for correlations based on themes for tenderness and flavor of which we followed suit. Pearson correlation coefficients are depicted in Tables 11 and 12. This study revealed moderate correlations between tenderness and juiciness at 0.593 and 0.519 for bulls and steers, respectively. These coefficients are of a similar order to findings by Legako et al. (2015) at *r* = 0.65. Furthermore, our findings for the correlations between myofibrillar force and intramuscular fat at *r* = −0.254 and −0.196 for bulls and steers respectively, are in line with those of Cafferky et al. (2019) at *r* = −0.26. The correlations shown between tenderness and myofibrillar force were *r* = −0.609 and −0.719 for bulls and steers respectively and between residual force and CT with *r* = −0.441 and −0.539 for bulls and steers, respectively. Lucero‐Borja et al. (2014) examined shear force values and tenderness between heifers of different slaughter weight as well as between heifers, steers, and cull cows of similar slaughter weights and found that heifers and steers of similar slaughter weight, aged 14 days postmortem, had similar WBSF and tenderness level with a correlation of *r* = −0.48, again demonstrating that findings herein concur with previously reported values. As expected, significantly (*p* < 0.01) high correlations were obtained for moisture and lipid content (*r* = −0.871 for bulls and *r* = −0.933 for steers). Residual and myofibrillar forces also showed highly significant (*p* < 0.01) correlations at 0.779 and 0.753 for bulls and steers respectively. Viewing the correlation results of traits associated with flavor (marbling, flavor and flavor intensity) and juiciness showed marbling was positively correlated with fat content (*r* = 0.678 and 0.737), flavor (*r* = 0.068 and 0.074), and flavor intensity (extremely low *r* = 0.003 and 0.001) for bulls and steers respectively. Jiu et al. (2020) furnished positive correlations between intramuscular fat% and flavor intensity approaching significance (*r* = 0.33, *p* = 0.0023). Legako et al. (2015) correlated fat percent from five USDA quality grades and obtained coefficients of 0.22, 0.29, and 0.27 with tenderness, juiciness, and flavor liking, respectively, and stated that overall liking was highly correlated with juiciness, tenderness and flavor liking (*p* < 0.001) indicating that consumers find it difficult to differentiate fully between attributes. Although our evidence here substantiates previous findings (Dikeman 1987; Thompson 2002; Serra et al. 2004) attesting to the fact that marbling and tenderness have a minor positive association (*r* = 0.138 and 0.111 for bulls and steers respectively), shifts in the palatability attributes have been emphasized by Corbin et al. (2015), O'Quinn et al. (2024) and Kerth et al. (2024). Corbin et al. (2015) provide correlations among consumer sensory scores for tenderness with juiciness, flavor, and fat with coefficients of 0.93, 0.88 and 0.81 respectively as well as juiciness and flavor at 0.87 and flavor and fat% at 0.74. These results are contrary to our findings. However, it is important to point out that further research is needed as indicated by Corbin et al. (2015), since it was unclear if the consumers were able to discern very tender beef or if superior juices and flavor in higher fat beef influenced their perception of tenderness due to the halo effect. Nonetheless, the aforementioned authors remark that it is plausible that differing consumer acceptability thresholds for tenderness exist across USDA quality grades. Consumers were more accepting of tenderness of higher marbling samples compared to lower marbling levels, despite efforts to reduce WBSF variation.

**TABLE 11:** Pearson correlation coefficients between certain physical, chemical and sensory characteristics of *Longissimus thoracis et lumborum* muscle from bulls.

	Marbling	Moisture	Tenderness	Connective tissue	Juiciness	Flavor	Flavor intensity	Myofibrillar force	Residual force
Moisture	−0.547**								
Tenderness	0.138	−0.155							
Connective tissue	0.151	−0.166	0.695**						
Juiciness	0.066	−0.065	0.539**	0.380**					
Flavor	0.068	−0.128	0.325**	0.062	0.423**				
Flavor intensity	0.003	−0.092	−0.047	0.323**	0.159	0.255*			
Myofibrillar force	−0.169	0.218*	−0,609**	−0.426**	−0.415	−0.204*	0.085		
Residual force	−0.219*	0.277**	−0.581**	−0.441**	−0.444	−0.345**	0.008	0.779**	
Lipid content	0.678**	−0.871**	0.203*	0.142	0.095	0.034	0.062	−0.254**	−0.302**

* *p* < 0.05.

** *p* < 0.01.

**TABLE 12:** Pearson correlation coefficients between certain physical, chemical and sensory characteristics of *Longissimus thoracis et lumborum* muscle from steers.

	Marbling	Moisture	Tenderness	Connective tissue	Juiciness	Flavor	Flavor intensity	Myofibrillar force	Residual force
Moisture	−0.685**								
Tenderness	0.111	−0.130							
Connective tissue	0.158	−0.182	0.750**						
Juiciness	0.026	0.054	0.519**	0.448**					
Flavor	0.074	−0.131	0.185*	0.129	0.344**				
Flavor intensity	0.001	−0.064	0.246*	0.278*	0.341**	0.49**			
Myofibrillar force	−0.146	0.197*	−0.719**	−0.571**	−0.298**	−0.117	−0.200*		
Residual force	−0.293	0.302*	−0.639**	−0.539**	−0.218**	−0.142	−0.206*	0.753**	
Lipid content	0.737**	−0.933**	0.119	0.159	−0.029	0.074	0.065	−0.196*	−0.317*

* *p* < 0.05.

** *p* < 0.01.

## 4 Conclusion

Young bulls of less than 15 months age were of similar eating quality to that of steers of the same age. Consumption of young bulls, particularly in North America, Australia, Ireland, and the United Kingdom should be considered with much greater versatility. In order to manage our food resources more favorably and sustainably, beef production systems using young bulls exhibiting higher production efficiencies will help to reduce GHG emissions; small‐framed animals being a prime example with a feeding regime that maintains a high growth rate throughout their life cycle. This would also lend itself to improved bull classification, animal welfare with cessation in castration, pain, and suffering of the animals and increased marketability for young bulls. More work is thus required to determine the suitability of consuming bull meat using consumer test measures in relation to palatability in these countries as well as educating the public about advantages of consuming bull beef.

## Author Contributions

**Yunus Khatri:** conceptualization (equal), data curation (equal), formal analysis (lead), funding acquisition (supporting), investigation (lead), methodology (equal), project administration (lead), writing – original draft (lead), writing – review and editing (lead). **Elisabeth Huff‐Lonergan:** conceptualization (equal), data curation (supporting), formal analysis (supporting), funding acquisition (lead), investigation (supporting), methodology (supporting), project administration (lead), supervision (lead), writing – review and editing (equal).

## Ethics Statement

The study was conducted according to the guidelines of the Declaration of Helsinki and approved by the Institutional Review Board of Iowa State University (Meat Lab. No. 09/1992).

## Consent

Informed consent was obtained from all subjects involved in the study.

## Conflicts of Interest

The authors declare no conflicts of interest.

## Supporting information


Appendix S1.

## Data Availability

The data that support the findings of this study are available from the corresponding author upon reasonable request.
