# Supplementary material for: Eating Quality of Ribeye Steaks From Young Bulls and Steers Is Comparable: A Study Examining the Effect of Sex, Frame Size, and Feed Time on Palatability Attributes
Source: Food Sci Nutr. 2025 Jun 30;13(7):e70359. doi: 10.1002/fsn3.70359 (PMC12208898; doi:10.1002/fsn3.70359)
Supplement: Supplementary file 1 — Appendix S1. [file FSN3-13-e70359-s001.docx]

Supporting Information

S1. Analysis of variance for *longissimus thoracis* muscle traits of bulls and steers

| Variable | Source | df | Sum of Squares | F-value | P>F |
| --- | --- | --- | --- | --- | --- |
| Fat thickness | Sex | 1 | 1.2180 | 65.85 | 0.0001 |
|  | Frame | 2 | 0.4882 | 13.20 | 0.0001 |
|  | Time | 2 | 0.8714 | 23.55 | 0.0092 |
|  | Sex x Frame | 2 | 0.1765 | 4.77 | 0.0001 |
|  | Sex x Time | 2 | 0.3097 | 8.37 | 0.0847 |
|  | Frame x Time | 4 | 0.0214 | 0.29 | 0.8395 |
| Lipid (%) | Sex | 1 | 93.5932 | 42.32 | 0.0001 |
|  | Frame | 2 | 43.8465 | 9.91 | 0.0001 |
|  | Time | 2 | 61.0807 | 13.81 | 0.0001 |
|  | Sex x Frame | 2 | 5.1813 | 1.17 | 0.3114 |
|  | Sex x Time | 2 | 11.2690 | 2.55 | 0.0802 |
|  | Frame x Time | 4 | 7.3811 | 0.83 | 0.5042 |
| Marbling Score | Sex | 1 | 3.959 X 10^5^ | 72.37 | 0.0001 |
|  | Frame | 2 | 1.280 X 10^5^ | 11.70 | 0.0001 |
|  | Time | 2 | 2.098 X 10^4^ | 1.92 | 0.1489 |
|  | Sex x Frame | 2 | 1.289 X 10^5^ | 1.18 | 0.3094 |
|  | Sex x Time | 2 | 2.649 X 10^5^ | 2.42 | 0.0907 |
|  | Frame x Time | 4 | 7.181 x10^4^ | 0.33 | 0.8599 |
| Moisture | Sex | 1 | 238.4509 | 48.74 | 0.0001 |
|  | Frame | 2 | 103.7523 | 10.60 | 0.0010 |
|  | Time | 2 | 45.4178 | 4.64 | 0.0104 |
|  | Sex x Frame | 2 | 10.3209 | 1.05 | 0.3497 |
|  | Sex x Time | 2 | 33.1609 | 3.39 | 0.0552 |
|  | Frame x Time | 4 | 12.0591 | 0.62 | 0.6513 |
| Tenderness | Sex | 1 | 3.4612 | 23.67 | 0.0001 |
|  | Frame | 2 | 2.0255 | 6.92 | 0.0012 |
|  | Time | 2 | 0.0490 | 0.17 | 0.8457 |
|  | Sex x Frame | 2 | 0.5069 | 1.73 | 0.1786 |
|  | Sex x Time | 2 | 0.1675 | 0.57 | 0.5646 |
|  | Frame x Time | 4 | 1.0683 | 1.83 | 0.1239 |
| Connective Tissue | Sex | 1 | 1.0601 | 8.97 | 0.0030 |
|  | Frame | 2 | 0.9301 | 3.94 | 0.0206 |
|  | Time | 2 | 0.2439 | 1.03 | 0.3575 |
|  | Sex x Frame | 2 | 0.6713 | 2.84 | 0.0600 |
|  | Sex x Time | 2 | 0.0585 | 0.25 | 0.7809 |
|  | Frame x Time | 4 | 1.4427 | 3.05 | 0.0174 |
| Juiciness | Sex | 1 | 1.9690 | 13.84 | 0.0002 |
|  | Frame | 2 | 0.2345 | 0.82 | 0.4397 |
|  | Time | 2 | 1.0581 | 3.72 | 0.0255 |
|  | Sex x Frame | 2 | 0.6019 | 2.12 | 0.1255 |
|  | Sex x Time | 2 | 0.2349 | 0.83 | 0.4391 |
|  | Frame x Time | 4 | 0.3185 | 0.56 | 0.6921 |

S1. Cont.

| Variable | Source | df | Sum of Squares | F-value | P>F |
| --- | --- | --- | --- | --- | --- |
| Flavor intensity | Sex | 1 | 1.6030 | 9.82 | 0.019 |
|  | Frame | 2 | 0.1608 | 0.49 | 0.611 |
|  | Time | 2 | 0.1917 | 0.59 | 0.556 |
|  | Sex x Frame | 2 | 0.3238 | 0.99 | 0.372 |
|  | Sex x Time | 2 | 0.2167 | 0.66 | 0.516 |
|  | Frame x Time | 4 | 1.3062 | 0.47 | 0.758 |
| Flavor | Sex | 1 | 0.5087 | 5.11 | 0.0246 |
|  | Frame | 2 | 0.2671 | 1.34 | 0.2634 |
|  | Time | 2 | 0.6368 | 3.20 | 0.0424 |
|  | Sex x Frame | 2 | 0.0189 | 0.10 | 0.9093 |
|  | Sex x Time | 2 | 0.0031 | 0.02 | 0.9847 |
|  | Frame x Time | 4 | 0.4549 | 1.14 | 0.3372 |
| Myofibrillar force | Sex | 1 | 10.8025 | 40.41 | 0.0001 |
|  | Frame | 2 | 1.3532 | 2.53 | 0.0814 |
|  | Time | 2 | 0.5920 | 1.11 | 0.3319 |
|  | Sex x Frame | 2 | 1.3166 | 2.46 | 0.0871 |
|  | Sex x Time | 2 | 1.1086 | 2.07 | 0.1277 |
|  | Frame x Time | 4 | 0.0893 | 0.08 | 0.9874 |
| Residual force | Sex | 1 | 7.7666 | 33.22 | 0.0001 |
|  | Frame | 2 | 2.0845 | 4.46 | 0.0124 |
|  | Time | 2 | 0.4469 | 0.96 | 0.3856 |
|  | Sex x Frame | 2 | 1.1218 | 2.40 | 0.0926 |
|  | Sex x Time | 2 | 0.6005 | 1.28 | 0.2787 |
|  | Frame x Time | 4 | 0.3439 | 0.37 | 0.8315 |

S2. Analysis of variance for *longissimus thoracis* muscle traits of bulls

| Variable | Source | df | Sum of Squares | F-value | P>F |
| --- | --- | --- | --- | --- | --- |
| Fat thickness | Frame | 2 | 0.0437 | 1.49 | 0.2291 |
|  | Time | 2 | 0.0759 | 2.59 | 0.0786 |
|  | Frame x Time | 4 | 0.078 | 1.33 | 0.2609 |
| Lipid (%) | Frame | 2 | 29.6364 | 7.60 | 0.0007 |
|  | Time | 2 | 23.1388 | 5.93 | 0.0034 |
|  | Frame x Time | 4 | 10.4699 | 1.34 | 0.2576 |
| Marbling Score | Frame | 2 | 4.40172 X 10^4^ | 5.22 | 0.0065 |
|  | Time | 2 | 1.9052 X 10^3^ | 0.23 | 0.7980 |
|  | Frame x Time | 4 | 1.0841 X 10^4^ | 0.64 | 0.6326 |
| Moisture | Frame | 2 | 25.0492 | 5.77 | 0.0039 |
|  | Time | 2 | 9.7275 | 2.24 | 0.1104 |
|  | Frame x Time | 4 | 7.5052 | 0.86 | 0.4873 |
| Tenderness | Frame | 2 | 0.2639 | 0.87 | 0.4208 |
|  | Time | 2 | 0.0929 | 0.31 | 0.7364 |
|  | Frame x Time | 4 | 0.7906 | 1.30 | 0.2714 |
| Connective Tissue | Frame | 2 | 0.2836 | 1.16 | 0.3151 |
|  | Time | 2 | 0.1817 | 0.75 | 0.4761 |
|  | Frame x Time | 4 | 2.2041 | 4.53 | 0.0018 |
| Juiciness | Frame | 2 | 0.0882 | 0.30 | 0.7428 |
|  | Time | 2 | 1.0253 | 3.46 | 0.0641 |
|  | Frame x Time | 4 | 0.2321 | 0.39 | 0.8142 |
| Flavor intensity | Frame | 2 | 0.0239 | 0.11 | 0.8969 |
|  | Time | 2 | 0.2310 | 1.05 | 0.3518 |
|  | Frame x Time | 4 | 1.7733 | 1.76 | 0.1400 |
| Flavor | Frame | 2 | 0.3117 | 0.39 | 0.6806 |
|  | Time | 2 | 0.3552 | 1.68 | 0.1906 |
|  | Frame x Time | 4 | 0.9725 | 0.62 | 0.6619 |
| Myofibrillar force | Frame | 2 | 0.2926 | 0.48 | 0.6182 |
|  | Time | 2 | 1.6278 | 2.68 | 0.0718 |
|  | Frame x Time | 4 | 0.2260 | 0.19 | 0.9452 |
| Residual force | Frame | 2 | 0.1417 | 0.30 | 0.7420 |
|  | Time | 2 | 0.9762 | 2.06 | 0.1314 |
|  | Frame x Time | 4 | 0.3061 | 0.32 | 0.8623 |

S3. Analysis of variance for *longissimus thoracis* muscle traits of steers

| Variable | Source | df | Sum of Squares | F-value | P>F |
| --- | --- | --- | --- | --- | --- |
| Fat thickness | Frame | 2 | 0.6246 | 14.07 | 0.0001 |
|  | Time | 2 | 1.1381 | 25.64 | 0.0001 |
|  | Frame x Time | 4 | 0.0120 | 0.14 | 0.9689 |
| Lipid (%) | Frame | 2 | 54.3027 | 9.41 | 0.0001 |
|  | Time | 2 | 74.6503 | 12.94 | 0.0001 |
|  | Frame x Time | 4 | 2.3781 | 0.21 | 0.9347 |
| Marbling Score | Frame | 2 | 9.6510 X 10^4^ | 7.21 | 0.0010 |
|  | Time | 2 | 4.6815 X 10^4^ | 3.5 | 0.0329 |
|  | Frame x Time | 4 | 1.1085 X 10^3^ | 0.04 | 0.9967 |
| Moisture | Frame | 2 | 40.1784 | 10.36 | 0.0001 |
|  | Time | 2 | 27.4693 | 7.09 | 0.0012 |
|  | Frame x Time | 4 | 3.7035 | 0.48 | 0.7521 |
| Tenderness | Frame | 2 | 2.2590 | 8.01 | 0.0005 |
|  | Time | 2 | 0.1227 | 0.44 | 0.6480 |
|  | Frame x Time | 4 | 0.5443 | 0.96 | 0.4291 |
| Connective Tissue | Frame | 2 | 1.3049 | 5.69 | 0.0042 |
|  | Time | 2 | 0.1189 | 0.52 | 0.5961 |
|  | Frame x Time | 4 | 0.1477 | 0.32 | 0.8626 |
| Juiciness | Frame | 2 | 0.7439 | 2.72 | 0.0691 |
|  | Time | 2 | 0.2558 | 0.94 | 0.3944 |
|  | Frame x Time | 4 | 0.6398 | 1.17 | 0.3262 |
| Flavor intensity | Frame | 2 | 0.2109 | 1.10 | 0.3355 |
|  | Time | 2 | 0.2832 | 1.48 | 0.2316 |
|  | Frame x Time | 4 | 0.1010· | 0.27 | 0.8997 |
| Flavor | Frame | 2 | 0.2841 | 0.85 | 0.4297 |
|  | Time | 2 | 0.0908 | 0.27 | 0.7625 |
|  | Frame x Time | 4 | 0.4845 | 0.72 | 0.5765 |
| Myofibrillar force | Frame | 2 | 2.3594 | 5.08 | 0.0074 |
|  | Time | 2 | 0.0543 | 0.12 | 0.8897 |
|  | Frame x Time | 4 | 0.0989 | 0.11 | 0.9801 |
| Residual force | Frame | 2 | 3.0493 | 6.61 | 0.0018 |
|  | Time | 2 | 0.0554 | 0.12 | 0.8688 |
|  | Frame x Time | 4 | 0.4527 | 0.49 | 0.7424 |
